# Importance of Community-Level Interventions During the COVID-19 Pandemic: Lessons from Sub-Saharan Africa

**DOI:** 10.4269/ajtmh.20-1533

**Published:** 2021-08-09

**Authors:** Ayomide Owoyemi, Elvis Anyaehiechukwu Okolie, Kasarachi Omitiran, Uchenna Anderson Amaechi, Babasola Olufemi Sodipo, Olufemi Ajumobi, Chukwudi Ernest Nnaji, Ijeoma Nkem Okedo-Alex

**Affiliations:** ^1^ Department of Biomedical and Health Information Sciences, University of Illinois, Chicago, Illinois;; ^2^ Teesside University, Middlesbrough, England;; ^3^ National Primary Health Care Development Agency (NPHCDA), Abuja, Nigeria;; ^4^ Strategy, Investment and Impact Division, Global Fund to Fight AIDS, Tuberculosis and Malaria, Geneva, Switzerland;; ^5^ Harvard T.H. Chan School of Public Health, Boston, Massachusetts;; ^6^ School of Community Health Sciences, University of Nevada, Reno, Nevada;; ^7^ School of Public Health and Family Medicine, University of Cape Town, Cape Town, South Africa;; ^8^ Department of Community Medicine, Alex Ekwueme Federal University Teaching Hospital Abakaliki, Nigeria

## Abstract

Community-level strategies are important in ensuring adequate control of disease outbreaks especially in sub-Saharan African countries. Learning from public health responses to previous infectious disease outbreaks is important in shaping these responses to COVID-19. This study aims to highlight and summarize the evidence from community-level interventions during infectious disease outbreaks in sub-Saharan Africa (SSA). We conducted a scoping review of published literature on community-level interventions and strategies adopted in different infectious disease outbreaks in SSA. To obtain relevant studies, we searched EMBASE, CINAHL, MEDLINE, and Google Scholar in August 2020. Our search was based on the combination of keywords such as coronavirus, flu, Ebola, community, rural, strategies, impact, effectiveness, feasibility, Africa, developing countries, and SSA. Studies that met the inclusion criteria were selected and synthesized under the following distinct themes: health education, sensitization, and communications; surveillance; and service delivery. Our review highlights community-based strategies that have been tried and tested with varying outcomes for different outbreaks in different sub-Saharan African communities, we believe they will inform the selection of strategies to adopt in managing the COVID-19 pandemic at the community level. The important aspects of these strategies were highlighted, requirements for successful implementation and the possible challenges that might be encountered were also discussed. Achieving control of the COVID-19 pandemic in sub-Saharan African communities, will require concerted community-based and community-led strategies, which in turn rely on the availability of necessary socioeconomic resources, and the contextual adaption of these interventions.

## INTRODUCTION

Globally, significant mortality and morbidity as a result of the COVID-19 pandemic have been documented in over 216 countries/territories of the world.^1^ It is important to note that communities are at the heart of the impact of the COVID-19 pandemic, which has drastically affected the daily lives, health, and socioeconomic status of communities in sub-Saharan Africa (SSA) with far-reaching consequences.^2^ Community-level interventions play a significant role in reducing person-to-person transmission and disease impact during an outbreak, thus, community participation is essential in the collective response to the coronavirus disease.^3^

Several non-pharmaceutical mitigation and control strategies have been put in place at the community level to control the spread of the virus. These non-pharmaceutical interventions include social strategies such as physical distancing, risk communication, and promotion as well as reinforcement of integrated water, sanitation, and hygiene practices in the community.^4^ These measures aim to prevent viral spread, and alleviate its effect in the short term, while long-term solutions such as vaccines are underway.^5^

Learning from public health responses to pandemics similar to the current outbreak of SARS-CoV2 and other infectious disease outbreaks in the past, is important in shaping the current response to COVID-19. Some of these other outbreaks in SSA will include viral epidemics due to Ebola virus, influenza, yellow fever, monkey pox, and other public health events such as tsetse fly invasions with associated human trypanosomiasis. Notably, the Ebola virus pandemic exposed the fragility of the health systems in the region and beamed the searchlight on the need to build resilient health systems that could prepare for, respond to, and withstand health crisis/shocks in a globally equitable and sustainable manner.^6^^–^^11^ A key feature of such resilient health systems, is the adoption of an integrated approach involving diverse actors and sectors with the community at the center because beyond the health sector, health-related events share a bidirectional relationship with non-health sectors such as agriculture, finance, water, and sanitation.^6^^,^^8^^,^^11^^,^^12^ As a part of building resilience, the implementation of lessons learnt in the sub-Saharan African region should be adapted to the contextual peculiarities of the subcontinent.^4^ The aim of this study therefore, was to review community-level responses to infectious disease outbreaks in SSA and highlight strategies that would be useful in managing the present COVID-19 pandemic.

## METHODS

In July–August 2020, we conducted a comprehensive search and review of published literature on community-level interventions and strategies adopted in infectious disease outbreaks, and other public health threats in SSA. We also summarized the measured outcomes associated with the reported interventions. Such outcomes included knowledge, behavior, perception, and practice. Relevant primary studies carried out in SSA, regardless of year of publication or study design, were included in the review. Studies not published in English language were excluded. The databases searched included MEDLINE, EMBASE, CINAHL, and Google Scholar. The search terms used included key words and Medical Subject heading (MeSH) terms such as COVID-19, coronavirus, flu, Ebola, smallpox, community, rural, interventions, strategies, impact, effectiveness, knowledge, acceptability, attitude, feasibility, practices, sub-Saharan Africa, Africa, and developing countries. The search terms were combined using the Boolean operators “AND” or “OR” and adapted for the various databases using appropriate syntaxes. The search strategy is presented in the Supplemental Appendix. These interventions were presented under the following distinct themes: health education, sensitization, and communications; surveillance; and service delivery. We reviewed 13 studies on community-based interventions implemented in SSA to curtail disease outbreaks with a focus on the problem addressed, intervention adopted, method of delivery, effectiveness of intervention, and documented challenges faced by such interventions.

## RESULTS

### Health education, sensitization, and communication.

#### Film-based community outreach (Republic of Congo).

The 2003 Human monkeypox outbreak in Impfondo town (Likouala region) coupled with high seroprevalence levels (23–83%) of monkeypox virus antibodies among community residents highlighted the need for public health action.^13^

Facilitating community involvement in monkeypox surveillance and providing monkeypox prevention were key intervention objectives. Films titled *Understanding Monkeypox* and *Monkeypox Testimonies* were produced. These films targeted a broad spectrum of monkeypox-related issues; modes of virus transmission, identification of monkeypox, disease consequences, prevention, and the need to seek care. The post-intervention survey revealed improvements in knowledge and positive health-related behavioral intents. For instance, health-seeking behavior measured by the willingness to take a family member with monkeypox to the hospital increased from 48% to 87%.^14^

The engagement of community leaders, involvement of community members as cast in the films and production of the films in the local language were vital to community participation. However, this intervention faced community mobilization challenges because of deeply rooted cultural beliefs and the paucity of transportation and communication infrastructure especially for hard-to-reach areas. Roess et al. highlighted the need for sustained health promotion efforts and encouragement of alternative practices that are culturally acceptable.^14^

#### Community-led infection prevention education (Cote d'lvoire).

Cote d'Ivoire’s proximity to the worst affected countries by Ebola necessitated a prevention plan. Gautier et al. reported that as part of the “National Plan on EVD Prevention and Response,” the government of Cote d'Ivoire in partnership with the International Rescue Committee (IRC), implemented a “community-led infection prevention” strategy in four selected districts: Biankouma, Danane, Odienne, and Touba.^15^ A “monitoring committee” made up of selected community members (community health workers, religious leaders, traditional healers, and women and youth leaders) was established during the intervention to sensitize the community residents.

A four-prong approach in sensitizing community members on “socially and culturally” appropriate Ebola virus disease (EVD) prevention practices was implemented. These comprised of; the review of EVD prevention messages in the context of prevailing local realities and recommendation of tolerable alternative practices, development and distribution of communication materials adapted to local needs, training of members of the “monitoring committees” to disseminate culturally appropriate EVD prevention information, and lastly, implementation of sensitization sessions near health centers and public settings, such as markets and from door to door.^15^

The intervention was credited with the increased practice of routine hand washing across the four selected districts, reduced consumption of bushmeat, and decreased incidence of unsafe practices relating to handshaking and burials. For instance, in 11 out of the 12 health areas where the intervention was implemented, participants reported compliance with majority of recommended practices. Despite documented constraints that included lack of supportive economic policies that offered alternative jobs and subsidy on other meat types, certain processes in the program design proved pivotal. The engagement of community leaders and recognizable individuals created program ownership and social acceptance of messages.^15^

#### Information dissemination.

Government officials in Liberia initially disseminated Ebola messaging through fliers, billboard advertisements, and radio messaging. Participants had concerns about the wording of message: “Ebola kills.” This approach fostered a sense of fear and contributed to the initial spread of distrust. In addition, the different health actors involved in the Ebola response crafted different and sometimes conflicting messages.^16^ These challenges were remedied by the use of community structures that used face-to-face communication between community members and members of the formal and informal health system, and dissemination of Ebola-related information in the local language through radios and trucks with speakers, which increased acceptability and health outcomes. It is worthy to mention that information passed on through community leaders was perceived to be more credible than information received from government health officials. This highlighted the significance of treating community members as integral health system actors, rather than passive recipients of health services.

#### Interpersonal communication.

Sierra Leone faced a significant proportion of the unprecedented mortality and morbidity outcomes after the 2014 EVD outbreak across West Africa.^17^ The impact of the EVD outbreak in Sierra Leone was further exacerbated by a spate of denials, uncertainty of the consequences after disease admission, and communication failure leading to distrust of the health system.^18^ Although mass media can reach a large number of individuals in a short time, it is not devoid of challenges; it may not feature options for dialogue, clarification seeking, and may lack an understanding of prevailing communication nuances in communities.^19^

Turay reports the outcome of a community-based interpersonal communication (IPC) intervention in Sierra Leone, directed at creating behavior change and ending disease transmission. The intervention recruited and trained members of the local community on disseminating EVD-related messages, precautionary guidelines, and health communication skills.^19^ Before the intervention, a spectrum of issues was observed; less than 45% of the community members acknowledged receiving and understanding EVD-related messages, there was high prevalence of resistance to recommended behavior change, difficulty in message personalization, misconceptions because of rumors, and the perceived lack of clarity in EVD messaging. Overall, the IPC intervention was considered successful as more than 90% of residents reported having better understanding of messages and were willing to practice recommended behaviors, residents became more tolerant and receptive to health workers, and increased confidence in case reporting by residents. Importantly, the impact of the IPC intervention was linked to factors such as the implementation of activities at locations and times convenient with residents, understanding of cultural values, interactive process of IPC interventions leading to trust building, engagement of opinion leaders, and the engagement of community members as project staff.

### Service delivery.

#### Community vaccination.

The Ebola disease control efforts in Congo, which include the ring vaccination strategy, were hampered by geographical and sociocultural challenges, which included community resistance and mistrust of health workers, especially in the rural communities.^20^ This necessitated the use of community vaccination strategy in complement with the ring strategy. Community vaccination involves vaccination of everyone in the neighborhood, or village, rather than vaccinating only the known contacts and contacts of contacts.^21^ This strategy was found to substantially speed up and enhance the probability of containment of the epidemic.^20^^,^^22^ The success of this strategy depends on the level of community accessibility and cooperation. This was overcome in the Congo through the engagement of locals for service delivery.^23^

#### Mental health services.

Evidence suggests that efforts directed at psychosocial support during epidemics are poorly implemented and devoid of best practices. In a Liberian town during the Ebola epidemic a “mental health and psychosocial (MPHSS)” response was implemented, the MPHSS intervention was aimed at raising EVD awareness and tackling apprehension arising from misconceptions.^24^ Furthermore, the impact of social exclusion experienced by Ebola survivors and relatives reinforced the need for reintegration activities and recovery efforts.

Before the commencement of psychosocial interventions, implementing agencies engaged community members to comprehend their experiences, assess psychosocial needs, and explore strategies for long-term community recovery. Prevalent “outbreak-related” stressors elicited include “feelings of fear, panic, lack of community-based support, loss of breadwinners, economic and food insecurity, increased conflict, and disrupted social cohesion.”^24^

This response was implemented using the Social Reconnection Groups (SRG) model. The SRG is a group model focused on improving the coping mechanisms of community members against daily stressors, promoting conflict resolution, and strengthening problem-solving processes.^24^^,^^25^ The SRG also provided a safe platform for the provision of appropriate EVD information and psychoeducation to highlight the uniqueness of individual coping mechanisms. The training of program staff and group facilitators, selection of SRG group members, and implementation of group sessions were core intervention activities. This approach will be more successful if it is delivered via multisectoral collaboration and active community participation.^24^

### Surveillance.

#### Community-based surveillance.

As the epidemic curve for the Ebola outbreak in Liberia rose, health workers were overwhelmed with the magnitude of contact tracing and surveillance that needed to be conducted; this posed a gap and a threat to the success of the outbreak response.^16^ This gap led to the formation of community-based surveillance teams by County Health Teams (CHTs) with the involvement of local NGOs. In some instances, the community was given the autonomy to determine the composition of these teams and even form burial teams as well.

Community members were tasked with the responsibility of keeping surveillance over homes to enable them to identify and report sick people to their local chief who in turn reported to the community health worker and then to the CHTs for evacuation to health facilities. Barker et al. noted that “…in general, health authorities identified and presented a problem to the community and delegated authority to the community to make a series of decisions on how to enact a solution.”^16^ The participatory nature of the community engagement used appeared quite effective, as the respondents of the study attribute its implementation to being critical to the containment of the Ebola outbreak in Liberia.

## DISCUSSION

Controlling any epidemic at the community level, requires well-implemented and relevant sensitization and education of community members with effective surveillance strategies that is integrated with and involves the community. With evidence showing correlations between behaviors in a community and the burden of epidemics, strategies that target these behaviors will be complementary in managing the COVID-19 pandemic especially in communities in SSA. These strategies include the use of films made in local languages, to improve awareness and knowledge of the disease in these communities especially in very remote areas. This mode of education was found to have positive effects on healthy behaviors and health-seeking behaviors. It also includes the use of community-led methods in creating and implementing prevention education that are context specific, with open information dissemination to members of the public in non-paternalistic ways.

These community-led disease prevention strategies must follow rigorous planning and implementation, underpinned by adherence to community participation principles. Such strategies are more likely to secure cooperation and ownership from the targeted communities, for example, the community-led tracking approach is credited as being critical to the containment of Ebola in Liberia. The use of community vaccination coupled with community-sensitive strategies will help ensure faster control of the epidemic. These strategies are not disparate solutions, they are complementary and might be more effective overall if they are integrated across the different communities. They also must be backed by appropriate considerations for socioeconomic and cultural factors to ensure people find them easier to embrace and adopt.

It is also important that these community-based efforts are linked with the respective Primary Healthcare (PHC) frameworks in those areas. The sustainability of these solutions is reliant on solid PHC foundations that can support these communities to continuously maintain these practices, long after the focus on the epidemic has reduced. In places where these PHC frameworks do not exist, this pandemic could be an opportunity to create building blocks for them; this will include the setting up of community champions, health influencers, ward health committees, and so on. These frameworks will help with the harmonization of these strategies as they will be more effective if they are deployed in a concerted way.

These strategies also highlight the relevance of an approach to infectious disease control and management beyond the usual framework of biological prevention and control, for example, the importance of mental health and psychosocial support during the pandemic to help community members cope better and have overall good health, is an important aspect that has not been well applied in communities. Although some of these interventions are non-pharmacological, they will be integral to the acceptance and widespread use of the COVID-19 vaccines in these communities. It is also important to conduct post-implementation assessments for these strategies to understand if they are effective. This will be relevant in their long-term adoption and create an evidence-based basis for integrating them into the disease response framework across Africa and elsewhere.

## CONCLUSION

Although it seems that the disease has been less virulent across the region in comparison to the Americas and Europe for different reasons, we are still in the middle of a pandemic and appropriately managing the outbreak with localized and suitable strategies will be essential to limiting the spread and subsequent mortalities from it. Successfully controlling the COVID pandemic in African communities will require concerted strategies that should involve community-based and community-led strategies targeted at behavioral change, surveillance, and service delivery. The success of these will be reliant on provision of necessary socioeconomic resources, alternatives, and contextualization for cultural and traditional practices.

## Supplemental Appendix


Supplemental materials
