# Supplementary material for: Importance of Community-Level Interventions During the COVID-19 Pandemic: Lessons from Sub-Saharan Africa
Source: Am J Trop Med Hyg. 2021 Aug 9;105(4):879–83. doi: 10.4269/ajtmh.20-1533 (PMC8592170; doi:10.4269/ajtmh.20-1533)
Supplement: Supplementary file 1 [file tpmd201533.SD1.docx]

**SUPPLEMENTAL APPENDIX**

| **Problem (P)** |  | **Interest/Intervention/Interest (I)** |  | **Outcome (O)** |  | **Settings (S)** |
| --- | --- | --- | --- | --- | --- | --- |
| Coronavirus OR Flu OR influenza OR Spanish Flu OR “COVID-19” OR SARS-CoV-2 OR Ebolavirus  OR  Smallpox | A  N  D | Community-based interventions” OR “Community Strategies” OR “Community-led Approaches” OR “Community” OR “Rural” OR Remote | A  N  D | “Impact” OR “Feasib*” OR “Effec*” OR “Knowledge” OR “Acceptability” OR “Attitudes” OR Perception OR “Practices” OR Manage OR management OR control OR Disease Control | A  N  D | Sub?Sahara* Africa * OR Africa OR “Sub?Saharan Africa” OR “Developing Countr*” OR “Low and Middle Income Countr*” OR “Low resource settings” |
